# Toll-Like Receptor 7 Agonists: Chemical Feature Based Pharmacophore Identification and Molecular Docking Studies

**DOI:** 10.1371/journal.pone.0056514

**Published:** 2013-03-20

**Authors:** Hui Yu, Hongwei Jin, Lidan Sun, Liangren Zhang, Gang Sun, Zhanli Wang, Yongchun Yu

**Affiliations:** 1 Department of Laboratory Centre, the Affiliated Tenth People's Hospital, Tongji University, Shanghai, China; 2 State Key Laboratory of Natural and Biomimetic Drugs, Peking University, Beijing, China; 3 The Second Affiliated Hospital, Baotou Medical College, Baotou, China; 4 The First Affiliated Hospital, Baotou Medical College, Baotou, China; Wake Forest University, United States of America

## Abstract

Chemical feature based pharmacophore models were generated for Toll-like receptors 7 (TLR7) agonists using HypoGen algorithm, which is implemented in the Discovery Studio software. Several methods tools used in validation of pharmacophore model were presented. The first hypothesis Hypo1 was considered to be the best pharmacophore model, which consists of four features: one hydrogen bond acceptor, one hydrogen bond donor, and two hydrophobic features. In addition, homology modeling and molecular docking studies were employed to probe the intermolecular interactions between TLR7 and its agonists. The results further confirmed the reliability of the pharmacophore model. The obtained pharmacophore model (Hypo1) was then employed as a query to screen the Traditional Chinese Medicine Database (TCMD) for other potential lead compounds. One hit was identified as a potent TLR7 agonist, which has antiviral activity against hepatitis virus *in vitro*. Therefore, our current work provides confidence for the utility of the selected chemical feature based pharmacophore model to design novel TLR7 agonists with desired biological activity.

## Introduction

Toll-like receptors (TLRs) are a family of highly conserved pattern recognition receptors (PRR) that can recognize pathogen-associated molecular patterns (PAMPs) present on or in bacteria, viruses, fungi and parasites [1]. Sensing these patterns by the TLRs initiates innate and adaptive immune responses against pathogens [2]. So far, thirteen TLRs have been reported [3]. The past 10 years have seen an explosion in the field of the TLRs, specifically the identification of novel TLR agonists. Agonists of the TLRs could enhance a specific immune response and have been proposed to be useful in battling cancer or infectious disease [4]. To date, several TLR agonists are in clinical development [5].

TLR7, one of the thirteen mammalian TLRs currently known, can be activated by specific small molecule agonists. Initially, imidazoquinoline derivatives including imiquimod and resiquimod were identified as TLR7 agonists [6]. In 2003, the guanosine analog loxoribine and the pyrimidine analog bropirimine were found to be TLR7 agonists [7]–[9]. Furthermore, many other research groups reported the discovery of novel TLR7 agonists. For example, Jones and co-workers reported TLR7 agonists and their use in the treatment of infectious diseases [10], [11]. Chong *et al*. reported novel phosphonic acid derivatives as new chemical leads for design of novel TLR7 agonists [12]. Cook *et al*. also discovered 8-oxoadenine derivative as TLR7 agonists [13]. Recently, Kurimoto *et al*. combined the adenine derivative with a labile carboxylic ester to identify new TLR7 agonist with antedrug characteristics [14]. Moreover, Pfizer reported the discovery of the deazapurine lead candidate compounds as TLR7 agonists [15]–[17]. However, administration of a TLR7 agonist is associated with some adverse events such as flu-like symptoms caused by induction of cytokines [18]. Therefore, the development of such kind of drug is focused on how to design more selective agonists.

Chemical feature based pharmacophore model may serve as a guide in the design of more potent ligands. Though many different TLR7 agonists had been synthesized and experimentally assessed, to the best of our knowledge, there is no information available regarding pharmacophore for such kind of compounds up to date. This study aims to construct the chemical feature based pharmacophore models for TLR7 agonists. A three-dimensional (3D) model of human TLR7 ligand-binding domain (LBD) was also constructed by means of a homology modeling procedure. At the same time, we reported the binding mode of human TLR7-LBD with agonists using docking method. Moreover, the obtained pharmacophore model was used as a query to retrieve TLR7 agonist candidates from the Traditional Chinese Medicine Database (TCMD) [19].

## Materials and Methods

All the calculations were performed in Discovery Studio program, version 2.1, from Accelrys (San Diego, USA).

### Pharmacophore model generation

**Figure 1:**
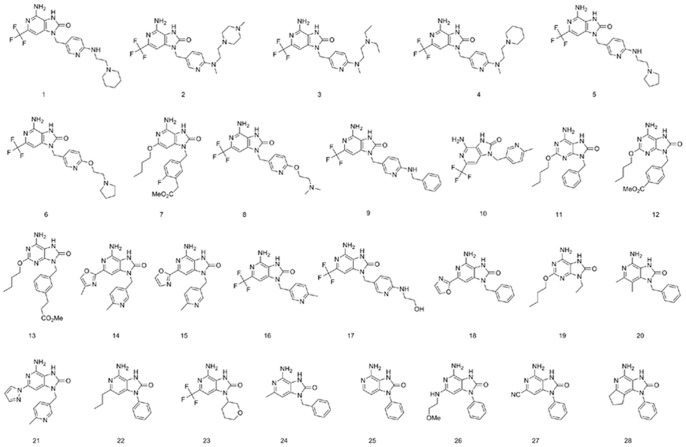
Chemical structures of the training set molecules applied to pharmacophore generation.

Twenty-eight compounds forming the training set were used to generate pharmacophore models. Structures were reported in Figure 1. The IUPAC names of the training set compounds were shown in supplementary information Text S1. An uncertainty factor of 3 for each compound was defined. Conformational analysis for each molecule was implemented using the Poling algorithm and the best quality generation method. A representative family of conformational models for each compound was generated specifying an energy threshold of 20 kcal/mol and the maximum number of conformations of 255. HypoGen algorithm was applied to build the 3D QSAR pharmacophore models [20]. In this study, hydrogen bond acceptor (HBA), hydrogen bond donor (HBD), and hydrophobic (HY) features were selected during the pharmacophore generation. The HypoGen algorithm was forced to find pharmacophores that contain at least one and at most two of every feature. All other parameters used were kept at their default settings. Also, the exclusion constraint feature was build based on the defined binding site as described below. The exclusion constraint feature represents an excluded volume in space, within a given radius. The combined exclusion constraint and chemical features should reduce the frequency of false positives in virtual screening.

### Validation of the pharamacophore model

The pharmacophore models were validated in three subsequent steps: cost analysis, Fischer's randomization test, and the test set prediction. First, HypoGen selects the best hypotheses by applying a cost analysis. There are three costs reported by HypoGen: the hypothesis cost, the fixed cost, and the null cost. If the difference between the cost of a hypothesis and the cost of the null hypothesis is greater than 60, there is an excellent chance the model represents a true correlation. If a hypothesis has a cost that differs from the null hypothesis by 40–60, there is a predictive correlation probability of 75%–90%. As the difference becomes less than 40, it may be difficult to find a predictive model. Second, statistical validation based on Fischer's randomization test was performed to assess the significance of a hypothesis generated by HypoGen. Desired confidence levels are 90%, 95%, 98%, and 99%. In our case, a statistical significance of 98% was allocated. Third, a test set of 28 diverse TLR7 agonists was selected to validate the best pharmacophore model. All test set compounds were built and minimized like all training set molecules. The relevant ligand-pharmacophore mappings were performed to estimate the predicted activity values of the compounds in test set.

### Sequence alignments and homology modeling of human TLR7-LBD

We developed structural models of cleaved TLR7-LBD by homology modeling as previously described [21]. In brief, amino acid sequence of human TLR7 ectodomain was extracted from TollML [22]. Five segments selected from four structures (PDB ID: 2Z80, 3CIG, 2Z64 and 2A0Z) were used as templates for human TLR7-LBD. The target-template alignment was carried out using the ClustalW program [23] and then manually refined based on the sequence alignment obtained from each LRR identified by TollML. The homology model of human TLR7-LBD was generated by the fully automated program MODELER. Loop regions of the protein structure were refined using Looper algorithm and CHARMm based molecular mechanics. The refined models were validated using PROCHECK [24] and ERRAT [25].

### Protein-protein docking

The validated model was then used for protein-protein docking to predict the homodimer of human TLR7-LBD. ZDOCK program was used to perform rigid body docking of two protein structures [26]. This protein-protein docking program rank the most probable predictions based on geometry, hydrophobicity and electrostatic complementarity of the molecular surface. In addition, it provides the option of reranking the docked poses with the ZRANK scoring function. In this study, angular step size for the rotational sampling of the ligand orientations was set to 15. The top 2,000 scoring poses yielded by ZRANK algorithm were produced. To select a model out from the top 2,000 scoring docked complexes, we applied several criteria, which were mentioned in the results and discussion sections. CHARMm energy minimization was performed for the final structure using RDOCK program.

### Binding site analysis

The Binding Site tools in Discovery Studio V2.1 software can be used to calculate, edit, partition, and display binding sites of a receptor. There are two site finding routines. One identifies cavities within the receptor using eraser algorithm [27], while the other finds sites as volume of selected ligand. In this study, the first method was used to identify the possible binding sites within homodimer of human TLR7-LBD. The standard default settings were used in the calculations except that minimum site size (points) was set to 500. The results can be used to guide the protein-ligand docking experiment.

### Protein-ligand docking

Docking calculations were carried out using the LibDock program [28]. LibDock uses protein site features referred to as HotSpots, consisting of two types (polar and apolar). The ligand poses are placed into the polar and aploar receptor interactions site. In this study, the MMFF force field was used for energy minimization of the ligand molecule. The binding sphere was initially defined as all residues of the target within 5 Å from the first binding site points as described above. Conformer Algorithm based on Energy Screening And Recursive build-up (CAESAR) was used for generating conformations. The Smart Minimizer was then used for *in-situ* ligand minimization. All other docking and subsequent scoring parameters used were kept at their default settings.

### Database search

The pharmacophore models can be used as queries to search 3D databases. In this study, TCMD built in the Catalyst data format containing approximately 10,458 compounds was used for virtual screening. We employed the first pharmacophore model (Hypo1) as a query against TCMD. A database search involves two methods: FAST and BEST. The FAST method computes already existing conformers of the database, while the BEST method is able to change the conformation of a molecule during computation. All queries were performed using the FAST approach. To be exported as a hit, the compounds must map the pharmacophore in all the features.

## Results and Discussion

### HypoGen model

**Figure 2:**
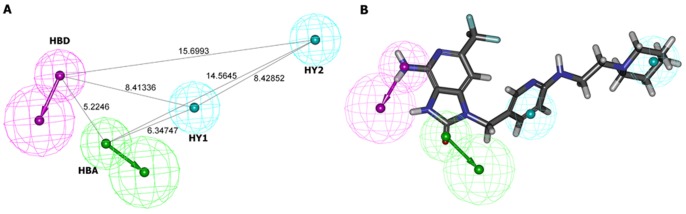
Pharmacophore model of TLR7 agonists generated by HypoGen. (A) Top scoring HypoGen pharmacophore Hypo1. (B) Hypo1 is aligned to the most active compound in the training set (EC_50_ = 0.2 nM). Pharmacophore features are color-coded (blue, hydrophobic; purple, hydrogen bond donor; green, hydrogen bond acceptor).

We selected 28 structures from different literatures as the training set for a HypoGen run. Their molecular structures were presented in Figure 1. The training set was selected by considering structural diversity and wide coverage of activity range (0.2 nM–3300 Mm). HypoGen exported the top 10 pharmacophore models (Table 1). All 10 pharmacophore models contain the same features: one HBA, one HBD, and two HY features. In this study, Hypo1 is the best pharmacophore model, characterized by the highest cost difference (62.505), best correlation coefficient (0.971), and lowest root-mean-square (RMS) deviation (0.584). Figure 2A showed the best pharmacophore model Hypo1. Output file of HypoGen run was shown in supplementary information Text S2.

**Table 1:** Output parameters of the top 10 hypotheses^a^.

Hypothesis	Correlation	RMS deviation	Total cost
1	0.971480	0.584417	115.082
2	0.970316	0.597810	115.548
3	0.971120	0.590975	115.551
4	0.964189	0.649014	115.666
5	0.965162	0.641724	115.759
6	0.963749	0.653023	115.761
7	0.964274	0.649204	115.826
8	0.960594	0.678931	116.008
9	0.965059	0.649011	116.736
10	0.954250	0.730628	117.101

a Null cost of 10 top-scored hypotheses is 177.587. Fixed cost value is 109.442. Configuration cost is 14.1361.

The mapping of Hypo1 onto one of the most active compound of the training set, compound 1 (EC_50_ = 0.2 nM), was shown in Figure 2B. The training set compound 1 fits very well all features of the pharmacophore model Hypo1. HBA is mapped by an 8-oxo group. HBD is mapped by a 6-amino group. The two hydrophobic features are fitted by two hydrophobic rings. Our results indicated that these moieties seemed to be essential for TLR7 agonistic activity.

### Evaluation of the HypoGen model

The cost parameters determine the quality of pharmacophore models. The difference between the total hypothesis cost and the null cost is of particular importance. Cost differences of 60 bits or higher lead to a predictive correlation probability of 90%. In this study, the null cost of the top 10 pharmacophore models is 177.587, the fixed cost value is 109.442, and configuration cost is 14.136 (Table 1). The difference between the first hypothesis (Hypo1) cost and the null cost is 62.505, which means that the pharmacophore model is deemed a 90% statistical probability. Moreover, the high correlation coefficient and the low RMS deviation indicated a reliable ability of this model to predict the training set compounds activities and confirmed that it was not generated by chance. Table 2 shows the actual and estimated activities of the 28 molecules from the training set based on the best pharmacophore model, Hypo1. As we can see from Table 2, most compounds were predicted correctly.

**Table 2:** Experimental biological data and estimated activity values of the training set molecules based on pharmacophore model Hypo1.

Compound	Measured activity^a^ (nM)	Estimated activity (nM)	Error factor^b^
1	0.20	0.45	+2.2
2	0.40	0.99	+2.5
3	1.00	1.40	+1.4
4	1.60	0.86	−1.9
5	1.70	1.40	−1.2
6	6.90	5.90	−1.2
7	26	77	+3
8	27	23	−1.2
9	31	33	+1.1
10	50	87	+1.7
11	63	77	+1.2
12	72	140	+2
13	87	84	−1
14	87	110	+1.3
15	100	120	+1.2
16	110	87	−1.2
17	120	65	−1.9
18	240	190	−1.3
19	260	200	−1.3
20	270	450	+1.7
21	630	140	−4.7
22	1100	2700	+2.5
23	1400	970	−1.5
24	1500	390	−3.9
25	2000	2800	+1.4
26	2200	770	−2.8
27	2200	2700	+1.2
28	3300	2500	−1.3

a References [10], [11], .

b The difference between estimated activity values and experimental activity values is represented as error (ratio between the estimated and experimental activity), with a negative sign if the actual activity is higher than that of the estimated.

**Figure 3:**
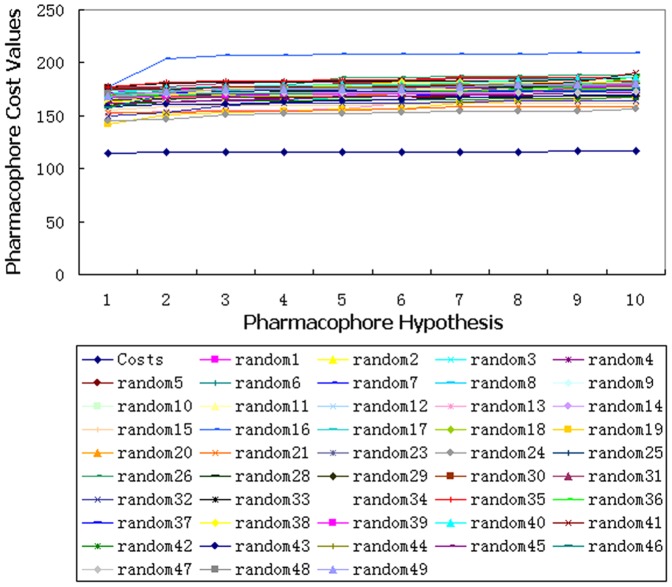
The difference in costs between the HypoGen runs and Fischer's randomization runs. The 98% confidence level was selected.

Fischer's randomization test is another approach to assess the statistical confidence of HypoGen models. Using the Fischer method, Discovery Studio randomly scrambled the activity values of all training set compounds and created 49 random spreadsheets. A HypoGen computation with each of them was performed by keeping the parameters of the initial HypoGen calculation. Our results showed that none of the exported hypotheses had a lower cost value than the initial hypothesis (Figure 3), indicating that there is a statistical significance level of 98% for Hypo1 to represent a true correlation in the training set activity data (Table S1).

**Figure 4:**
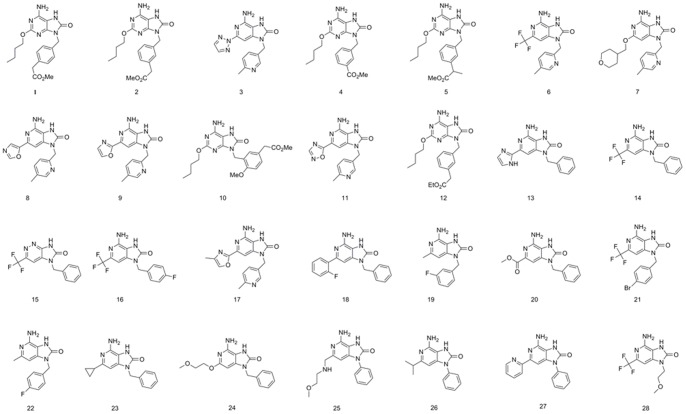
Chemical structures of the test set for validation of the predictive power of pharmacophore model Hypo1.

Furthermore, a test set containing 28 TLR7 agonists of different activity classes was analyzed to check the predictive power of the best pharmacophore model Hypo1 (Figure 4). The IUPAC names of the test set compounds were shown in supplementary information Text S3. The molecules and the corresponding conformational models were edited using the same method as for the training set compounds. In the test set analysis, most of the EC_50_ values were predicted correctly. Out of the 28 measured activity values, 25 were predicted with an error factor less than 5, and 1 was predicted with an error factor less than 10. The 2 remaining estimations were carried out with an error factor below 15. The results were presented in Table 3.

**Table 3:** Experimental biological data and estimated activity values of the test set molecules based on pharmacophore model Hypo1.

Compound	Measured activity^a^ (nM)	Estimated activity (nM)	Error factor^b^
1	10	89	+8.9
2	50	62	+1.2
3	51	173	+3.4
4	52	75	+1.4
5	75	67	−1.1
6	95	71	−1.3
7	99	76	−1.3
8	100	295	+3
9	104	118	+1.1
10	108	71	−1.5
11	127	141	+1.1
12	188	14	−13.4
13	215	173	−1.2
14	252	105	−2.4
15	269	105	−1.2
16	302	79	−3.8
17	355	108	−3.3
18	450	134	−3.4
19	453	101	−4.5
20	497	127	−3.9
21	656	170	−3.9
22	926	224	−4.1
23	933	439	−2.1
24	955	91	−10.5
25	1300	1175	−1.1
26	1600	3202	+2
27	1710	2858	+1.7
28	2002	7815	+3.9

a References [10], .

b The difference between estimated activity values and experimental activity values is represented as error (ratio between the estimated and experimental activity), with a negative sign if the actual activity is higher than that of the estimated.

### Homology modeling of human TLR7-LBD

**Figure 5:**
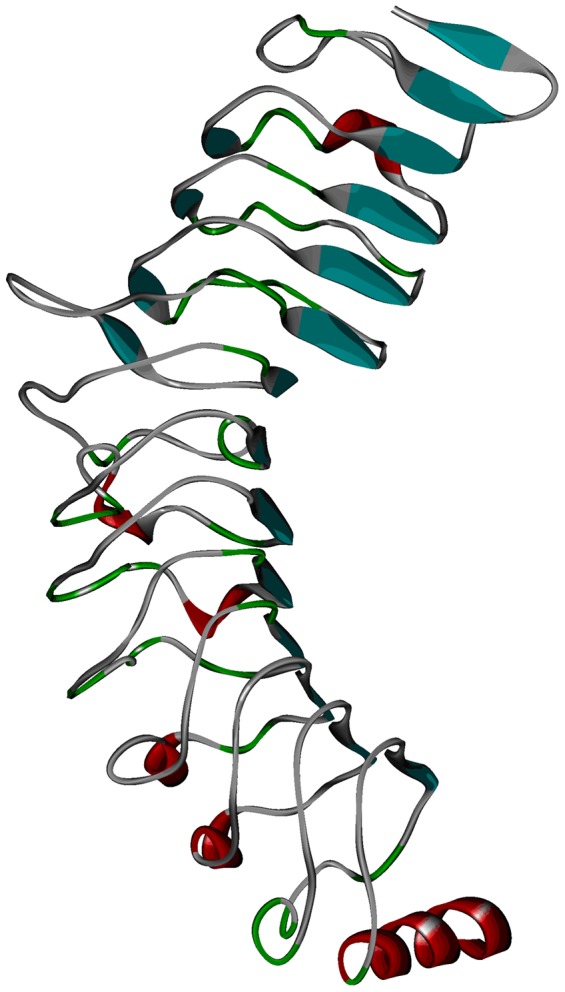
Structural model of human TLR7-LBD.

Leucine-rich repeats (LRRs) are protein-ligand interaction domains found in a variety of proteins. The structures of TLRs consist of a LRR ectodomain, a transmembrane helix, and a cytoplasmic Toll/IL-1 receptor homology (TIR) signaling domain. To date, several crystal structures of TLR ectodomains have been determined, including human TLR1/2/3/4 and mouse TLR3/4. By comparing these structure-known TLRs, TLR7 has a longer amino acid sequence. It also contains an irregular segment between its LRR14 and LRR15. Therefore, these structure-known TLRs are not suitable to serve as a full length template. Recent studies showed that TLR7 ectodomain was cleaved in the endolysosome to recognize ligands [29]. Visintin et al. also found that N-terminal portion of TLR7 is necessary for function but not ligand binding [30]. Therefore, structural model of cleaved TLR7-LBD could be used to predict possible configurations of the receptor-ligand complex. In order to build TLR7-LBD homology model, LRR segments with higher sequence similarity to the individual LRRs in TLR7 were selected from the four structure-known TLRs as previously described [21]. The segments were then served as the multiple templates. The 3D coordinates of the models were created by MODELER, and modified by Looper algorithm. The final model was subjected to CHARMm energy minimization. Figure 5 showed the structural model of TLR7-LBD.

The quality of the refined model was further assessed by ERRAT and PROCHECK programs. The ERRAT score evaluates the quality of a protein structure by considering non-bonded atomic interactions, and a score of greater than 50 is acceptable. The TLR3 and our refined model yielded ERRAT scores of 80.682 and 76.716, respectively. The evaluation results were clearly well within the range of good quality. PROCHECK program gives another assessment criterion by analyzing residue-by-residue geometry and overall structural geometry. The Ramachandran plot of the X-ray crystal structure of TLR3 showed that 75.5% of the residues were in the most favored regions, 24.2% in additional allowed regions, and 0.3% in generously allowed regions (Figure S1). All the residues of our refined model were also found in the allowed regions: 73.2% of the residues in the most favored regions, 25.5% in additional allowed regions, and 1.3% in generously allowed regions (Figure S1).

Recent studies showed that TLR exists as a monomer in solution, and dimerization only takes place after ligand binding. Therefore, we modeled human TLR7-LBD homodimer through protein-protein docking methods. In order to attempt to set up a reliable theoretical method to predict the TLR homodimer interaction, protein-protein docking method (ZDOCK) was used. To confirm the accuracy of this method, we firstly performed rigid-body docking for the crystal structure of TLR3 homodimer. The native TLR3 homodimer structure was reproduced and presented in the top 10 solution of ZDOCK. The results verify the reliability of ZDOCK in TLR-TLR docking calculations. Therefore, we used this method in our subsequent TLR7-TLR7 docking.

**Figure 6:**
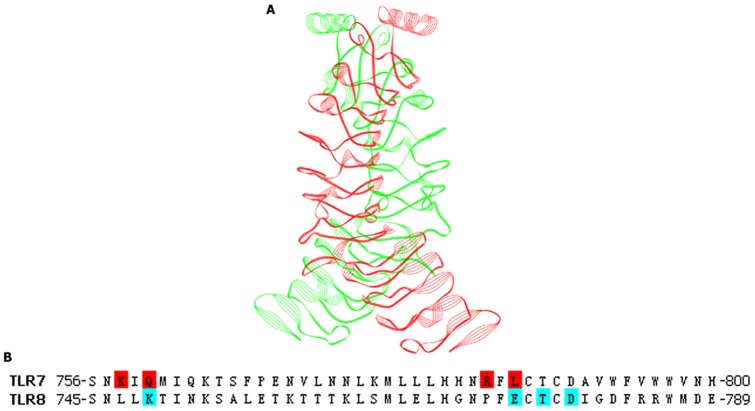
Human TLR7-LBD homodimer model observed in the docking methods. (A) One monomer is colored in green (A chain) and the other in red (B chain). (B) The residues involved in protein-protein interfaces.

Moreover, TLR8 and TLR7 were found to be closely related because of their intracellular localization and nucleic acid ligand [31]. We therefore used the recently published predicted structure of the TLR8 homodimer as a guide to perform protein-protein docking study [32]. Sequence alignment of TLR7 and TLR8 was shown in Figure S2. The residues involved in TLR8 dimerization are located at the C-terminus (Lys749, Glu775, Thr777, Asp779, Ser805, Gly807, and Arg810). We therefore suppose that the corresponding residues in TLR7 might contribute to protein-protein interaction. We then used ZDOCK program in our protein-protein docking calculations. From the results models, we chose the final docked complex based upon the predicted protein-protein interfaces as described above. The constructed homodimer model was refined by energy minimization, and the resulting homodimer model was shown in Figure 6A. As shown in Figure 6B, the residues involved in TLR8 dimerization were shaded in cyan, and the corresponding residues in TLR7-LBD were shaded in red.

### Protein-ligand docking studies

Following development of the model, we first analyzed human TLR7-LBD homodimer model to identify the ligand binding site. Seven active sites were obtained, and the locations of these sites in human TLR7-LBD homodimer model are shown in Figure S3. The previous studies suggested that several residues were essential for the ligand recognition, including Lys502, Ser504, Gly526, Gln531, Asn551, Arg553, Leu556, Ser575 and His578 [21]. It was reported that TLR9's Asp535 was determined to be required for the TLR9 function [33]. In addition, the mutant of TLR8's Asp543 that corresponds to TLR9's Asp535 abolished the TLR8 function [34]. Because the TLR7/8/9 are highly homologous, TLR7 might have a ligand-binding region located spatially around the Arg553 residue that corresponds to TLR9's Asp535 and TLR8's Asp543. It is obvious that the site 1 (blue region) in this study was in agreement with the results described above. This site in our model was surrounded mainly by the residues Ala482, Lys502, Asn503, Ser504, Phe506, Gly526, Leu528, Ser530, Gln531, Thr550, Asn551, Asn552, Arg553, Leu556, Ser575, Asn576, His578 and Gln581. Based on our theoretical results, this site is therefore chosen to perform the docking studies.

**Figure 7:**
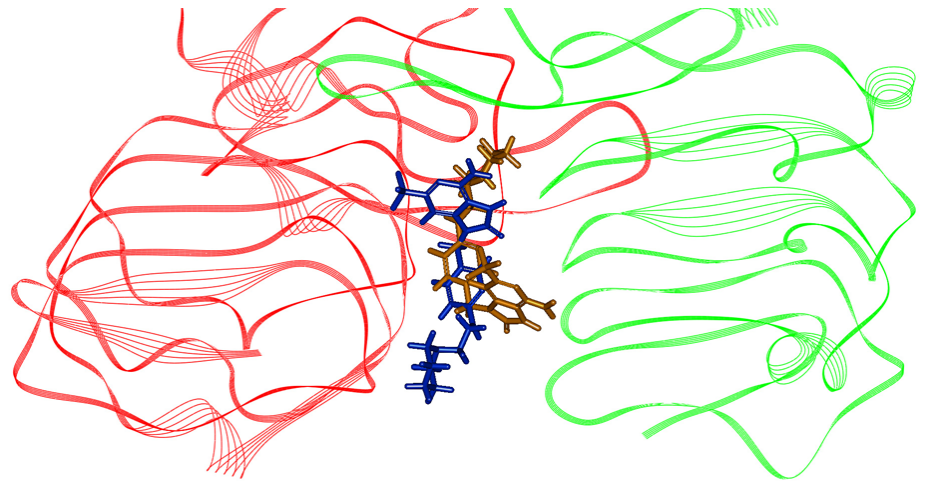
Docking conformations of human TLR7-LBD homodimer model bound compound 1 in the training set. Conformations of compound 1 are color-coded (pose1, orange;pose2, blue).

In order to understand the ligand recognition in TLR7 signaling, we initially carried out docking with the most active compound of the training set, compound 1. The docking program produces 94 poses with different orientations within the defined active site. Cluster analysis basing on RMS deviation was then performed to investigate the distribution of different binding modes. These docking poses could roughly be divided into two distinct groups. Two typical poses (pose1 and pose2) were then selected for further investigations. Figure 7 showed that compound 1 was placed in two different and opposite orientations. We selected pose1 as the final complex of TLR7-LBD based on the scoring functions such as LibDockScore, −PLP1, -PLP2, Jain, and Ludi as shown in Table 4.

**Table 4:** Docking scores of two typical poses of compound 1.

	−PLP1	−PLP2	Jain	LibDockScore	Ludi
Pose 1	79.10	79.01	2.37	100.74	307
Pose 2	67.84	67.65	1.87	94.97	225

**Figure 8:**
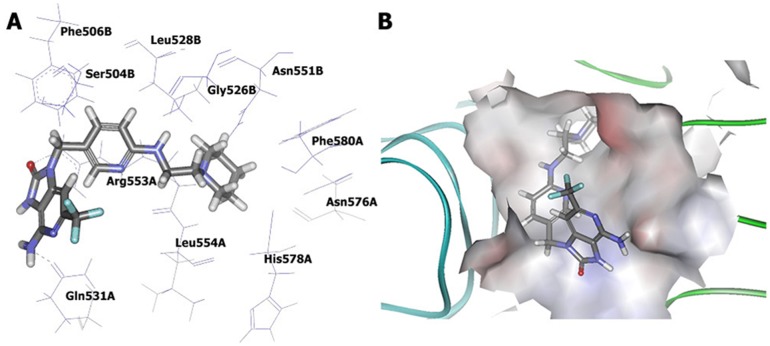
Interaction between human TLR7-LBD homodimer model and compound 1 in the training set as predicted by molecular docking. (A) Binding mode of compound 1. The hydrogen bonds are labeled by black lines. (B) The surface of human TLR7 binding site is color-coded by electrostatic potential (blue, positive charge; white, neutral; red, negative charge).

**Figure 9:**
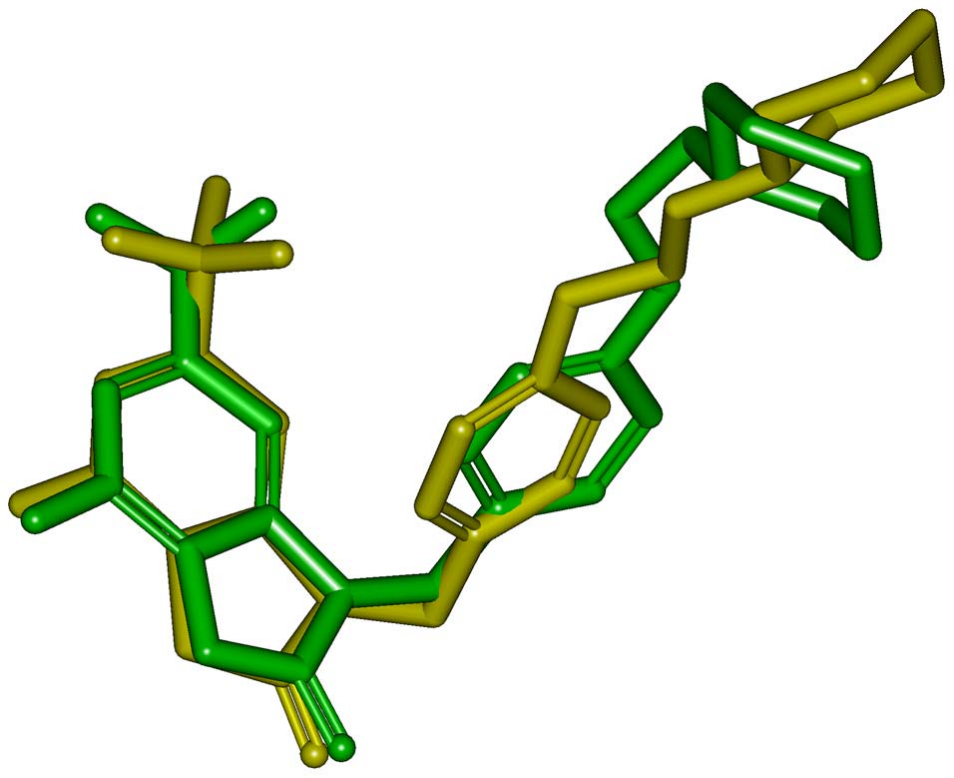
The docking conformation of compound 1 in the training set (green) was compared with that of the optimized one generated by pharmacophore model (yellow).

Possible mode of compound 1 and its interaction with human TLR7-LBD was shown in Figure 8A. Several residues are essential for complex formation: Ser504, Phe506, Gly526, Leu528, Gln531, Asn551, Arg553, Leu554, Asn576 and His578. This result was consistent with previous reports [21], which also found that most of these residues were critical for ligand binding. Inspecting the model structure, we know that several hydrogen bonds are formed between compound 1 and human TLR7-LBD. Interestingly, the 6-amino group of compound 1 was predicted to interact with Gln531 and the 8-oxo group was predicted to interact with Arg553. It became obvious that compound 1 satisfied the expected hydrogen bond interactions as defined by Hypo1. The modeling also suggested that the hydrophobic groups of compound 1 contacted with a hydrophobic region, which is comprised of the side chains of Phe506, Gly526, Leu528, Leu554, and Phe580. These hydrophobic interactions between compound 1 and human TLR7-LBD also satisfied the expected hydrophobic features as defined by Hypo 1. Figure 8B illustrated the electrostatic potential surface of TLR7 ligand-binding cavity. It is interesting to observe that the ligand-binding cavity of human TLR7 was an area of neutral charge (white), which implicated that compound 1 maintained the hydrophobic interaction as observed in the docking study. Moreover, the pharmacophore model derived conformation and the docking conformation of compound 1 were superposed with a RMS deviation value of 1.53 (Figure 9). This result further confirmed that the specific interaction between human TLR7-LBD and compound 1 was consistent with that proposed by the phamacophore.

To further evaluate the binding characteristics, other nine TLR7 agonists (compound 2–10) of the training set were docked into human TLR7-LBD model. The numbers of docking poses for compound 2–10 were 89, 96, 98, 100, 97, 89, 96, 100 and 79, respectively. The docking poses were then analysed and classified, and the final docked protein-ligand complexes were selected as described for compound 1. Interestingly, it has been proposed that these TLR7 agonists docked into the binding pockets in a similar orientation, establishing hydrogen bond interactions with protein residues Gln531 and Arg553 (Figure S4). In addition, hydrophobic groups of compound 2, 3, 4, 5, 6, 8 and 9 interacted with residues Phe506, Gly526, Leu528, Leu554, and Phe580. However, due to the lack of one hydrophobic feature (HY2), the hydrophobic groups of compound 7 and 10 only contacted with Phe506, Gly526 and Leu528. Leu554 and Phe580 did not participate in these hydrophobic interactions. The binding modes of these nine TLR7 agonists were agreement with that proposed by pharmacophore model. We, next, calculate the RMS deviation between the final docking conformation and the pharmacophore model derived conformation to quantify structural similarity (Figure S4). As shown in results, RMS deviation of each docked pose to its corresponding pharmacophore model derived structure is <2 Å. The results further validate the ability of the pharmacophore model to identify active conformation of TLR7 agonists.

### Virtual screening of novel TLR7 agonist candidates

The pharmacophore model Hypo1 was used in a 3D database query to find new structures for design of TLR7 agonists. Hypo1 captured 1853 hits from the entire TCMD (10,458) compounds. A problem related to this pharmacophore model is obviously the selectivity in filtering TCMD compounds, which may be considered as being low. Some compounds may be captured because of the small extensive spatial demands of a four-feature pharmacophore model. In general, 3D queries containing locations of pharmacophore features as well as restrictions on shape imposed by specifying excluded volumes may be useful for reducing false positive detections. An additional volume constraint was therefore added to the query (Figure S5). Excluded volume spheres were positioned coincident with atoms of human TLR7-LBD complex that were within 5 Å of binding site. The radii of the spheres were set to 1.2 Å. The calculated model was then used to filter 1853 hits captured by Hypo1. The query identified 6 hits. Those hits satisfied both the volume constraint and chemical features.

**Figure 10:**
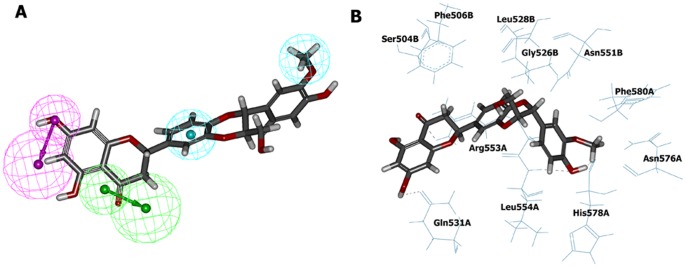
Hit compounds identified by pharmacophore screening. (A) Hit compound (Compound_Number_7720) is aligned to pharmacophore model Hypo1. Pharmacophore features are color-coded (blue, hydrophobic; purple, hydrogen bond donor; green, hydrogen bond acceptor). (B) Interaction between human TLR7-LBD homodimer model and hit compound as predicted by molecular docking. The hydrogen bonds are labeled by black lines.

Among these 6 hits, one compound (Compound_Number_7720) satisfied the demands of Lipinski ‘rule of five’ (Figure 10), which appears possible to identify compounds having ‘drug-like’ properties. This hit was then docked into the TLR7-LBD binding site. Figure 10 shows a possible energy-minimized docking model of TLR7–hit compound. The docking results showed that the hydrogen-bond acceptor feature of hit compound was predicted to interact with Gln531, and the hydrogen-bond donor feature was predicted to interact with Arg553. Also, there are additional two hydrogen-bonds seen in the complex. In addition, the hydrophobic groups of hit compound satisfy the expected hydrophobic interactions as defined by pharmacophore model. Moreover, a marked similarity was observed between the hit compound binding features in the docking model and that proposed by the pharmacophore model with a RMS deviation value of 1.95 Å (Figure S6). Interestingly, the biological activity of this hit compound was evaluated *in vitro*, which demonstrated anti-hepatitis virus activities [19]. Therefore, the compound was a lead candidate structure for design of novel TLR7 agonists.

## Conclusions

This study represents the first successful attempt to obtain a pharmacophore model Hypo1 that defines the pharmacophoric requirements for TLR7 agonistic activity. The validation results provide additional confidence in the proposed pharmacophore model. Our current model can be utilized as a guide for future studies to design the structurally novel TLR7 agonists. We also presented for the first time the study of binding mode between the active compounds of the training set and human TLR7-LBD homodimer model using the docking method. The predicted ligand-binding residues in TLR7 were in agreement with other studies. The docking result further validated the robustness of the obtained pharmacophore. Moreover, the utility of Hypo1 to perform virtual screening in TCMD is shown that the model was able to identify a lead candidate, which can be used as a starting scaffold for design of novel TLR7 agonists.

## Supporting Information

Figure S1 **Ramachandran plots qualities.** (A) Ramachandran plot of TLR3 (PDB ID: 1ZIW). (B) Ramachandran plot of the homology model of human TLR7-LBD. The shading on the plot represents the different regions (red, the most favored regions; yellow, the allowed regions; beige, the generously allowed regions; and white, the disallowed regions).
(PDF)

Figure S2 **The sequence of TLR7 was compared with the sequence of TLR8 by CLUSTAL W program.** The shading on the sequence represents the sequence similarity (dark blue, identical; blue, strong; cyan, weak, white, non-matching).
(PDF)

Figure S3 **The possible binding sites of human TLR7-LBD homodimer model.** Binding sites are color-coded (Site 1, blue; Site 2, orange; Site 3, red; Site 4, purple; Site 5, cyan, Site 6, yellow; Site 7, green).
(PDF)

Figure S4 **Binding modes of nine compounds in the training set.** (A) Hypo1 is aligned to nine compounds in the training set. (B) Interactions between human TLR7-LBD homodimer model and nine compounds in the training set as predicted by molecular docking. The hydrogen bonds are labeled by black lines. (C) The docking conformations of nine compounds in the training set (green) were compared with those generated by pharmacophore model (yellow). RMS deviation values of compound 2–10 were 1.97, 1.86, 1.67, 1.98, 1.91, 1.53, 1.89, 1.86, and 1.48 Å, respectively.
(PDF)

Figure S5 **The combined excluded volumes and hypothesis used as a query to search 3D database.**
(PDF)

Figure S6 **The docking conformation of hit compound (green) was compared with that of the optimized one generated by pharmacophore model (yellow).**
(PDF)

Table S1 **The difference in correlation values between the HypoGen runs and Fischer's randomization runs. The 98% confidence level was selected.**
(CSV)

Text S1 **The IUPAC names of the training set compounds.**
(DOC)

Text S2 **Output file of HypoGen run.**
(LOG)

Text S3 **The IUPAC names of the test set compounds.**
(DOC)
