# Supplementary figures and images for: Toll-Like Receptor 7 Agonists: Chemical Feature Based Pharmacophore Identification and Molecular Docking Studies
Source: PLoS One. 2013 Mar 20;8(3):e56514. doi: 10.1371/journal.pone.0056514 (PMC3603940; doi:10.1371/journal.pone.0056514)

**A**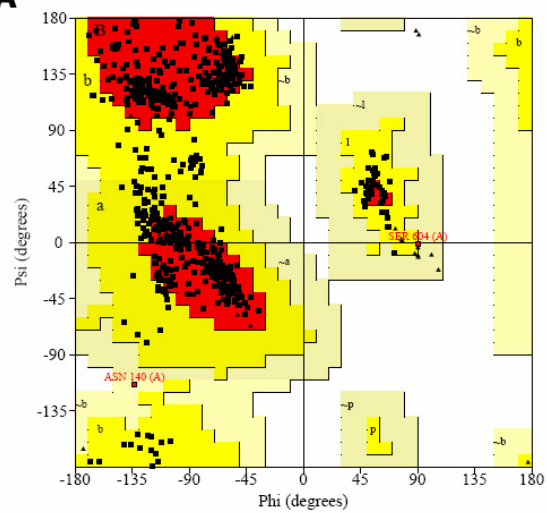**B**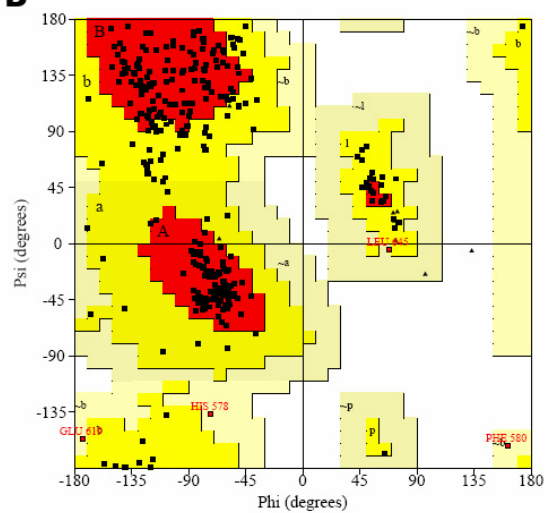

Supplement: Figure S1 — Ramachandran plots qualities. (A) Ramachandran plot of TLR3 (PDB ID: 1ZIW). (B) Ramachandran plot of the homology model of human TLR7-LBD. The shading on the plot represents the different regions (red, the most favored regions; yellow, the allowed regions; beige, the generously allowed regions; and white, the disallowed regions). (PDF) [file pone.0056514.s001.pdf]

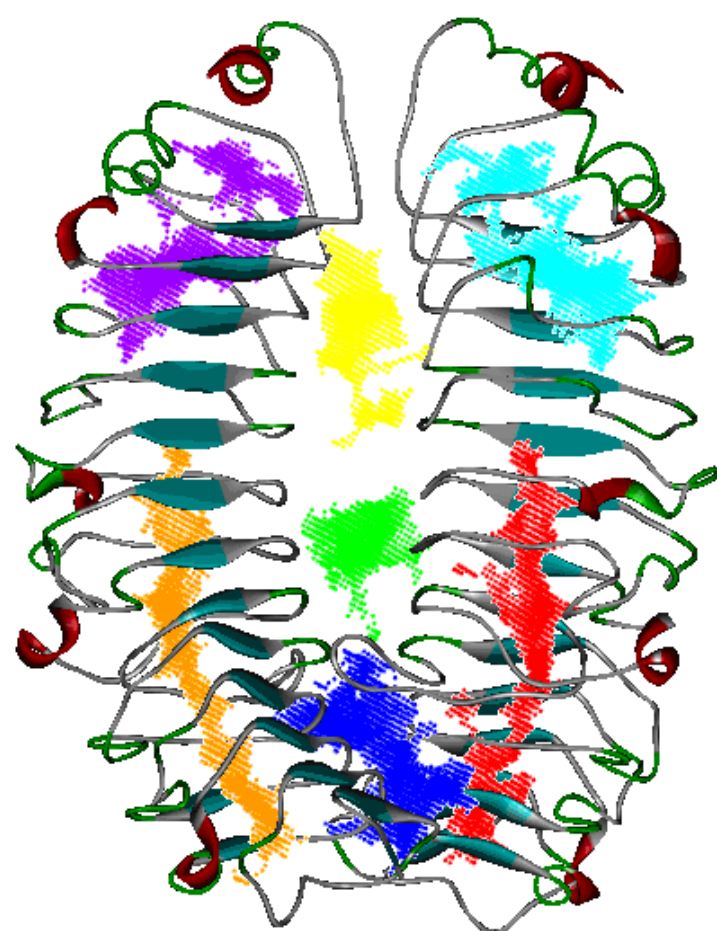

Supplement: Figure S3 — The possible binding sites of human TLR7-LBD homodimer model. Binding sites are color-coded (Site 1, blue; Site 2, orange; Site 3, red; Site 4, purple; Site 5, cyan, Site 6, yellow; Site 7, green). (PDF) [file pone.0056514.s003.pdf]

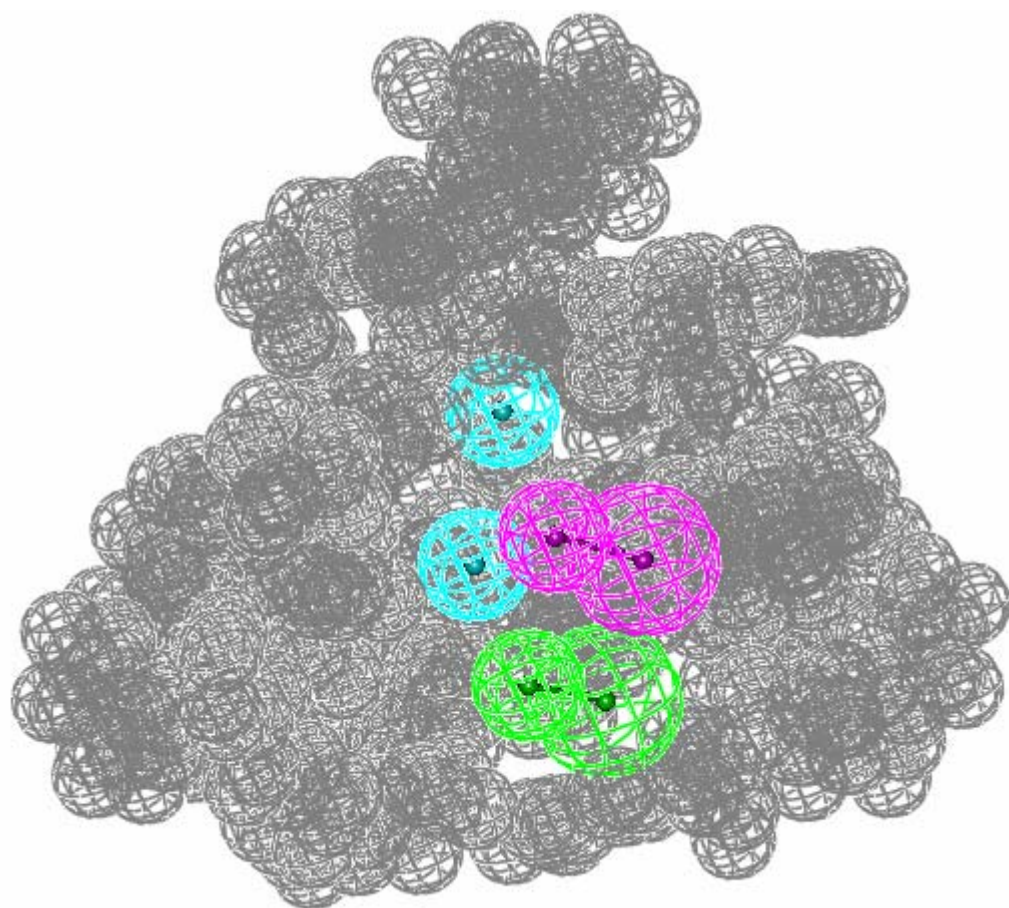

Supplement: Figure S5 — The combined excluded volumes and hypothesis used as a query to search 3D database. (PDF) [file pone.0056514.s005.pdf]

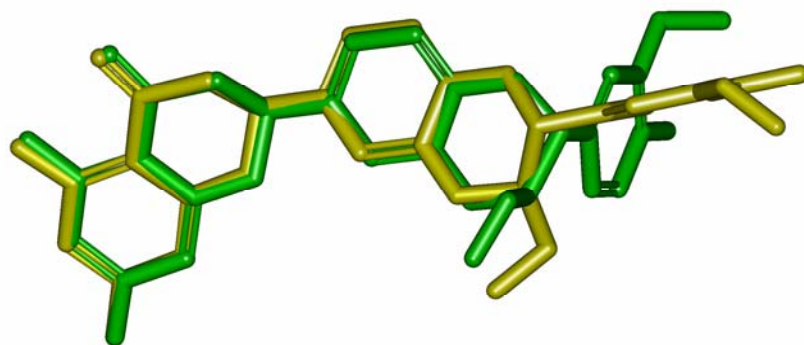

Supplement: Figure S6 — The docking conformation of hit compound (green) was compared with that of the optimized one generated by pharmacophore model (yellow). (PDF) [file pone.0056514.s006.pdf]
