# Supplementary material for: Toll-Like Receptor 7 Agonists: Chemical Feature Based Pharmacophore Identification and Molecular Docking Studies
Source: PLoS One. 2013 Mar 20;8(3):e56514. doi: 10.1371/journal.pone.0056514 (PMC3603940; doi:10.1371/journal.pone.0056514)
Supplement: Figure S2 — The sequence of TLR7 was compared with the sequence of TLR8 by CLUSTAL W program. The shading on the sequence represents the sequence similarity (dark blue, identical; blue, strong; cyan, weak, white, non-matching). (PDF) [file pone.0056514.s002.pdf]

```
tlr7 MVFPMWTLKRQILILFNIILISKLLGARWFPKTLPCDVTLDVPKNHVIVDCTDKHLTEIPGGIPTNTTNLTLTINHIPDISPASFHRLDHLVEIDFRCNCVPIPLGSKNNMCIKRLQIK
tlr8 MENMFLQSSMLTCIFLLISGSCELCAEENFSRSYPCDE--KKQNDSVIAECSNRRLQEVPQTVGKYVTELDLSDNFITHITNESFQGLQNLTKINLNHNPNVQHQNGNPGIQSNGLNIT

tlr7 PRSFSGLTYLKSLYLDGNQLLEIPQGLPPSLQLLSLEANNIFSIRKENLTELANIEILYLGQNCYYRNPCYVSYSIEKDAFLNLTKLKVLSLKDNNVTAVPTVLPSTLTELYLYNNMIA
tlr8 DGAFLNLKNLRELLLEDNQLPQIPSGLPESLTELSLIQNNIYNITKEGISRLINLKNLYLAWNCYFNKVCEKTN-IEDGVFETLTNLELLSLSFNSLSHVPPKLPSSLRKLFLSNTQIK

tlr7 KIQEDDFNNLNQLQILDLSGNCPRCYNAPFPCAPCKNNSPLQIPVNAFDALTELKVLRLHSNSLQHVPPRWFKNINKLQELDLSQNFLAKEIGDAKFLHFLPSLIQLDLSFNFELQVYR
tlr8 YISEEDFKGLINLTLLDLSGNCPRCFNAPFPCVPCDGGASINIDRFAFQNLTQLRYLNLSSTSLRKINAAWFKNMPHLKVLDLEFNYLVGEIASGAFLTMLPRLEILDLSFNYIKGSYP

tlr7 ASMNLSQAFSSLKSLKILRIRGYVFKELKSFNLSPLHNLQNLEVLDLGTNFIKIANLSMFKQFKRLKVIDLSVNKISPSGDSSEVGFCSNARTSVESYEPQVLEQLHYFRYDKYARSCR
tlr8 QHINISRNFSKLLSLRALHLRGYVFQELREDDFQPLMQLPNLSTINLGINFIKQIDFKLFQNFSNLEIIYLSENRISPLVKDTRQSYANSSSFQRHIRK----RRSTDFEFDPHSNFYH

tlr7 FKNKEASFMSVNESCYKYGQTLDLSKNSIFFVKSSDFQHLSFLKCLNLSGNLISQTLNGSEFQPLAELRYLDFSNNRLDLLHSTAFEELHKLEVLDISSNSHYFQSEGITHMLNFTKNL
tlr8 FTRP----L-IKPQCAAYGKALDLSLNSIFFIGPNQFENLPDIACLNLSANSNAQVLSGTEFSAIPHVKYLDLTNNRLDFDNASALTELSDLEVLDLSYNSHYFRIAGVTHHLEFIQNF

tlr7 KVLQKLMMNDNDISSSTSRT-MESESLRTLEFRGNHLDVLWREGDNRYLQLFKNLLKLEELDISKNSLSFLPSGVFDGMPPNLKNLSLAKNGLKSFSWKKLQCLKNLETLDLSHNQLTT
tlr8 TNLKVLNLSHNNIYTLTDKYNLESKSLVELVFSGNRLDILWNDDDNRYISIFKGLKNLTRLDLSLNRLKHIPNEAFLNLPASLTELHINDNMLKFFNWTLLQQFPRLELLDLRGNKLLF

tlr7 VPERLSNCSRSLKNLILKNNQIRSLTKYFLQDAFQLRYLDLSSNKIQMIQKTSFPENVLNNLKMLLLHHNRFLCTCDAVWFVWWVNHTEVTIPYLATDVTCVGPGAHKGQSVISLDLYT
tlr8 LTDSLSDFTSSLRTLLLSHNRISHLPSGFLSEVSSLKHLDLSSNLLKTINKSALETKTTTKLSMLELHGNPFECTCDIGDFRRWMDEHLNVKIPRLVDVICASPGDQRGKSIVSLELTT

tlr7 CELDLTNLILFSLSISVSLFLMVMMTASHLYFWDVWYIYHFCKAKIKGYQRLISPDCCYDAFIVYDTKDPAVTEWVLAELVAKLEDPREKHFNLCLEERDWLPGQPVLENLSQSIQLSK
tlr8 CVSDVTAVILFFFTFFITTMVMLAALAHHLFYWDVWFIYNVCLAKVKGYRSLSTSQTFYDAYISYDTKDASVTDWVINELRYHLEESRDKNVLLCLEERDWDPGLAIIDNLMQSINQSK

tlr7 KTVFVMTDKYAKTENFKIAFYLSHQRLMDEKVDVIILIFLEKPFQKSKFLQLRKRLCGSSVLEWPTNPQAHPYFWQCLKNALATDNHVAYSQVFKETV---
tlr8 KTVFVLTKKYAKSWNFKTAFYLALQRLMDENMDVIIFILLEPVLQHSQYLRLRQRICKSSILQWPDNPKAEGLFWQTLRNVVLTENDSRYNNMYVDSIKQY
```
