# Supplementary material for: Toll-Like Receptor 7 Agonists: Chemical Feature Based Pharmacophore Identification and Molecular Docking Studies
Source: PLoS One. 2013 Mar 20;8(3):e56514. doi: 10.1371/journal.pone.0056514 (PMC3603940; doi:10.1371/journal.pone.0056514)
Supplement: Figure S4 — Binding modes of nine compounds in the training set. (A) Hypo1 is aligned to nine compounds in the training set. (B) Interactions between human TLR7-LBD homodimer model and nine compounds in the training set as predicted by molecular docking. The hydrogen bonds are labeled by black lines. (C) The docking conformations of nine compounds in the training set (green) were compared with those generated by pharmacophore model (yellow). RMS deviation values of compound 2–10 were 1.97, 1.86, 1.67, 1.98, 1.91, 1.53, 1.89, 1.86, and 1.48 Å, respectively. (PDF) [file pone.0056514.s004.pdf]

A

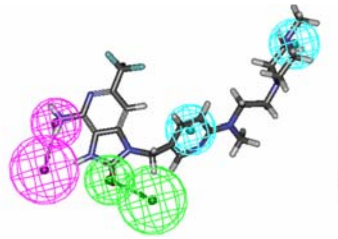

Compound2

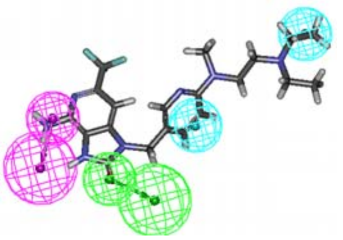

Compound3

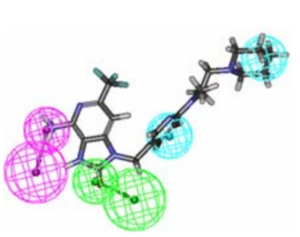

Compound4

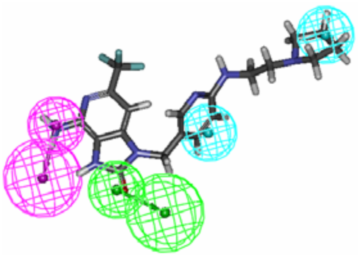

Compound5

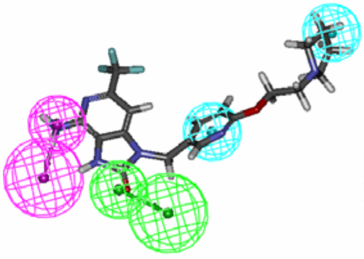

Compound6

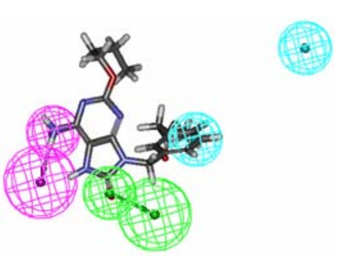

Compound7

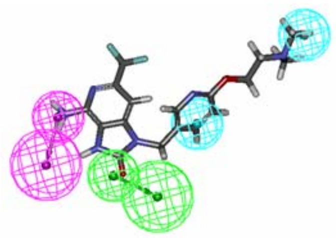

Compound8

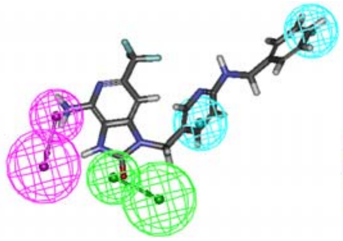

Compound9

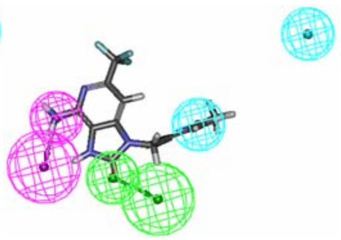

Compound10

B

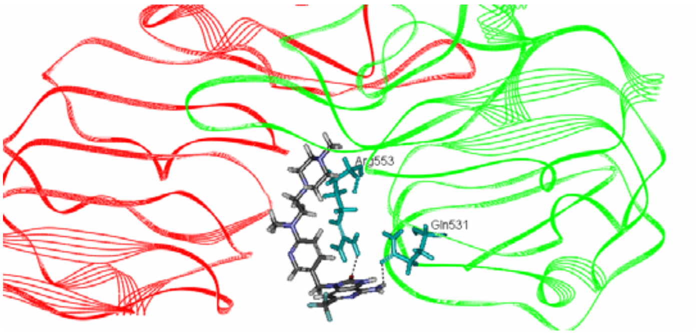

Compound2

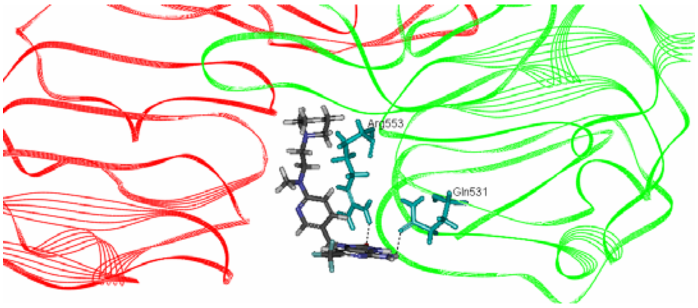

Compound3

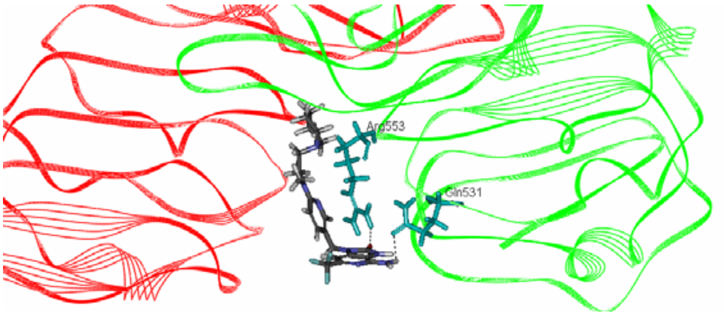

Compound4

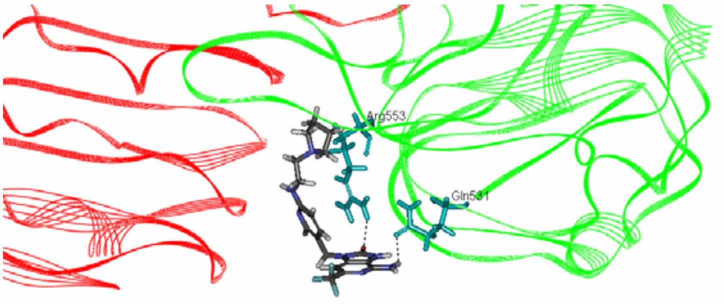

Compound5

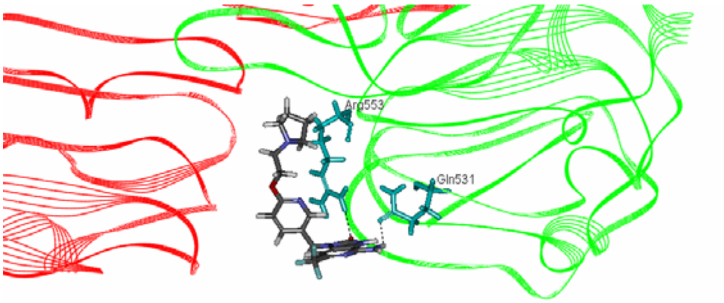

Compound6

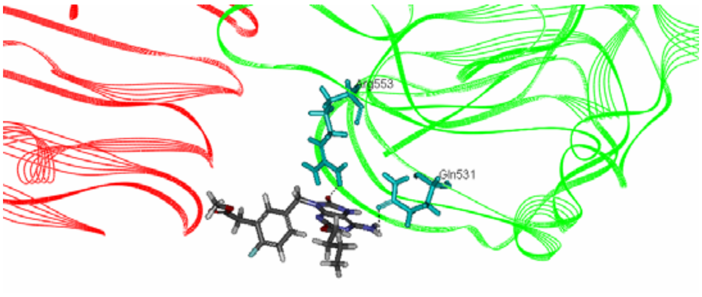

Compound7

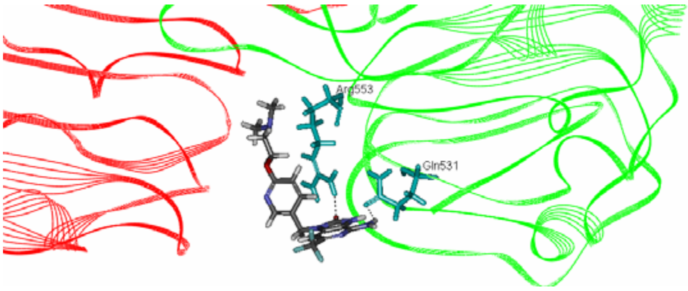

Compound8

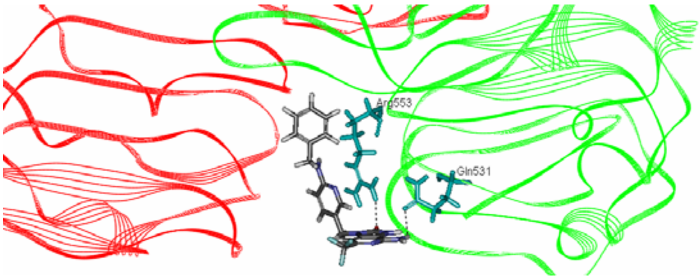

Compound9

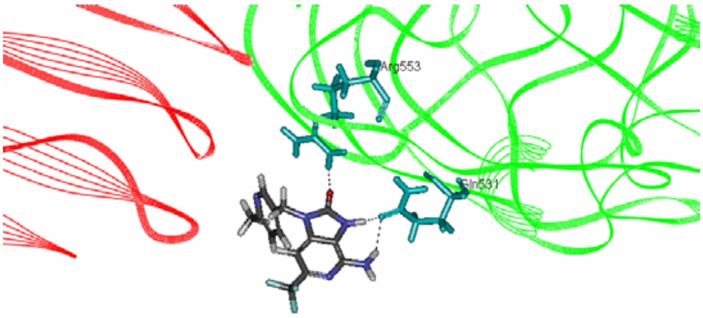

Compound10

C

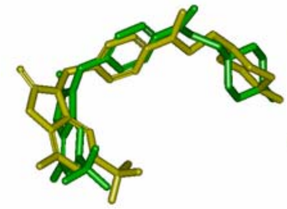

Compound2

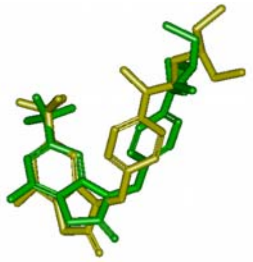

Compound3

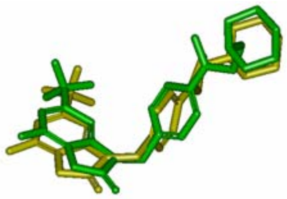

Compound4

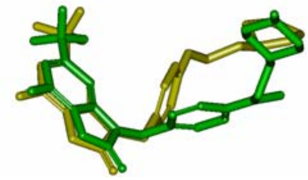

Compound5

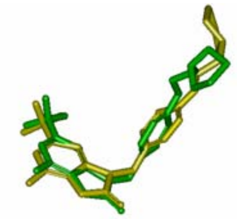

Compound6

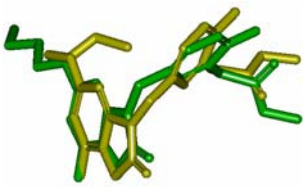

Compound7

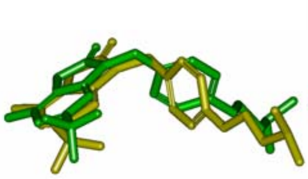

Compound8

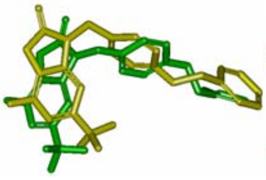

Compound9

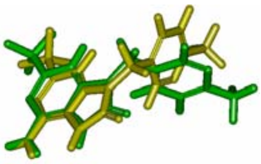

Compound10
