# Supplementary material for: Toll-Like Receptor 7 Agonists: Chemical Feature Based Pharmacophore Identification and Molecular Docking Studies
Source: PLoS One. 2013 Mar 20;8(3):e56514. doi: 10.1371/journal.pone.0056514 (PMC3603940; doi:10.1371/journal.pone.0056514)
Supplement: Text S1 — The IUPAC names of the training set compounds. (DOC) [file pone.0056514.s008.doc]

**The IUPAC names of training set compounds**

**Compound 1.**

4-amino-1-((6-(2-(piperidin-1-yl)ethylamino)pyridin-3-yl)methyl)-6-(trifluoromethyl)-1H-imidazo[4,5-c]pyridin-2(3H)-one

**Compound 2.**

4-amino-1-((6-(methyl(2-(4-methylpiperazin-1-yl)ethyl)amino)pyridin-3-yl)methyl)-6-(trifluoromethyl)-1H-imidazo[4,5-c]pyridin-2(3H)-one

**Compound 3.**

4-amino-1-((6-((2-(diethylamino)ethyl)(methyl)amino)pyridin-3-yl)methyl)-6-(trifluoromethyl)-1H-imidazo[4,5-c]pyridin-2(3H)-one

**Compound 4.**

4-amino-1-((6-(methyl(2-(piperidin-1-yl)ethyl)amino)pyridin-3-yl)methyl)-6-(trifluoromethyl)-1H-imidazo[4,5-c]pyridin-2(3H)-one

**Compound 5.**

4-amino-1-((6-(2-(pyrrolidin-1-yl)ethylamino)pyridin-3-yl)methyl)-6-(trifluoromethyl)-1H-imidazo[4,5-c]pyridin-2(3H)-one

**Compound 6.**

4-amino-1-((2-(2-(pyrrolidin-1-yl)ethoxy)pyridin-3-yl)methyl)-6-(trifluoromethyl)-1H-imidazo[4,5-c]pyridin-2(3H)-one

**Compound 7.**

Methyl(5-((6-amino-2-butoxy-8-oxo-7,8-dihydro-9H-purin-9-yl)methyl)-2-fluorophenyl)acetate

**Compound 8.**

4-amino-1-((6-(2-(dimethylamino)ethoxy)pyridin-3-yl)methyl)-6-(trifluoromethyl)-1H-imidazo[4,5-c]pyridin-2(3H)-one

**Compound 9.**

4-amino-1-((6-(benzylamino)pyridin-3-yl)methyl)-6-(trifluoromethyl)-1H-imidazo[4,5-c]pyridin-2(3H)-one

**Compound 10.**

4-amino-1-((6-methylpyridin-3-yl)methyl)-6-(trifluoromethyl)-1H-imidazo[4,5-c]pyridin-2(3H)-one

**Compound 11.**

6-amino-9-benzyl-2-butoxy-7H-purin-8(9H)-one

**Compound 12.**

Methyl 4-((6-Amino-2-butoxy-8-oxo-7,8-dihydro-9H-purin-9-yl) methyl)benzoate

**Compound 13.**

Methyl 3-{3- ((6-Amino-2-butoxy-8-oxo-7,8-dihydro-9H-purin-9-yl ) methyl)phenyl)propanoate

**Compound 14.**

4-amino-6-(4-methyloxazol-2-yl)-1-((6-methylpyridin-3-yl)methyl)-1H-imidazo[4,5-c]pyridin-2(3H)-one

**Compound 15.**

4-amino-1-((6-methylpyridin-3-yl)methyl)-6-(oxazol-2-yl)-1H-imidazo[4,5-c]pyridin-2(3H)-one

**Compound 16.**

4-amino-1-((6-methylpyridin-2-yl)methyl)-6-(trifluoromethyl)-1H-imidazo[4,5-c]pyridin-2(3H)-one

**Compound 17.**

4-amino-1-((6-(2-hydroxyethylamino)pyridin-3-yl)methyl)-6-(trifluoromethyl)-1H-imidazo[4,5-c]pyridin-2(3H)-one

**Compound 18.**

4-amino-1-benzyl-6-(oxazol-2-yl)-1H-imidazo[4,5-c]pyridin-2(3H)-one

**Compound 19.**

6-amino-2-butoxy-9-ethyl-7H-purin-8(9H)-one

**Compound 20.**

4-amino-1-benzyl-6,7-dimethyl-1H-imidazo[4,5-c]pyridin-2(3H)-one

**Compound 21.**

4-amino-1-((6-methylpyridin-3-yl)methyl)-6-(1H-pyrazol-1-yl)-1H-imidazo[4,5-c]pyridin-2(3H)-one

**Compound 22.**

4-amino-1-phenyl-6-propyl-1H-imidazo[4,5-c]pyridin-2(3H)-one

**Compound 23.**

4-amino-1-((tetrahydro-2H-pyran-3-yl)methyl)-6-(trifluoromethyl)-1H-imidazo[4,5-c]pyridin-2(3H)-one

**Compound 24.**

4-amino-1-benzyl-6-methyl-1H-imidazo[4,5-c]pyridin-2(3H)-one

**Compound 25.**

4-amino-1-phenyl-1H-imidazo[4,5-c]pyridin-2(3H)-one

**Compound 26.**

4-amino-6-(2-methoxyethylamino)-1-phenyl-1H-imidazo[4,5-c]pyridin-2(3H)-one

**Compound 27.**

4-amino-2-oxo-1-phenyl-2,3-dihydro-1H-imidazo[4,5-c]pyridine-6-carbonitrile

**Compound 28.**

4-amino-1-phenyl-3,6,7,8-tetrahydrocyclopenta[b]imidazo[4,5-d]pyridin-2(1H)-one
