# Supplementary material for: Toll-Like Receptor 7 Agonists: Chemical Feature Based Pharmacophore Identification and Molecular Docking Studies
Source: PLoS One. 2013 Mar 20;8(3):e56514. doi: 10.1371/journal.pone.0056514 (PMC3603940; doi:10.1371/journal.pone.0056514)
Supplement: Text S3 — The IUPAC names of the test set compounds. (DOC) [file pone.0056514.s010.doc]

**The IUPAC names of test set compounds**

**Compound 1.**

Methyl(4-((6-Amino-2-butoxy-8-oxo-7,8-dihydro-9H-purin-9-yl)methyl)phenyl)acetate

**Compound 2.**

Methyl(3-((6-Amino-2-butoxy-8-oxo-7,8-dihydro-9H-purin-9-yl)methyl)phenyl)acetate

**Compound 3.**

4-amino-1-((6-methylpyridin-3-yl)methyl)-6-(2H-1,2,3-triazol-2-yl)-1H-imidazo[4,5-c]pyridin-2(3H)-one

**Compound 4.**

Methyl 3-((6-Amino-2-butoxy-8-oxo-7,8-dihydro-9H-purin-9-yl)methyl)benzoate

**Compound 5.**

Methyll2-(3- ((6-Amino-2-butoxy- 8-oxo-7, 8- dihydro-9H-purin-9-yl)methyl)phenyl)propanoate

**Compound 6.**

4-amino-1-((5-methylpyridin-2-yl)methyl)-6-(trifluoromethyl)-1H-imidazo[4,5-c]pyridin-2(3H)-one

**Compound 7.**

4-amino-1-((5-methylpyridin-2-yl)methyl)-6-((tetrahydro-2H-pyran-4-yl)methoxy)-1H-imidazo[4,5-c]pyridin-2(3H)-one

**Compound 8.**

4-amino-1-((5-methylpyridin-2-yl)methyl)-6-(oxazol-5-yl)-1H-imidazo[4,5-c]pyridin-2(3H)-one

**Compound 9.**

4-amino-1-((5-methylpyridin-2-yl)methyl)-6-(oxazol-2-yl)-1H-imidazo[4,5-c]pyridin-2(3H)-one

**Compound 10.**

methyl 2-(3-((6-amino-2-butoxy-8-oxo-7H-purin-9(8H)-yl)methyl)-4-methoxyphenyl)acetate

**Compound 11.**

4-amino-1-((6-methylpyridin-3-yl)methyl)-6-(1,2,4-oxadiazol-5-yl)-1H-imidazo[4,5-c]pyridin-2(3H)-one

**Compound 12.**

ethyl 2-(3-((6-amino-2-butoxy-8-oxo-7H-purin-9(8H)-yl)methyl)phenyl)acetate

**Compound 13.**

4-amino-1-benzyl-6-(1H-imidazol-2-yl)-1H-imidazo[4,5-c]pyridin-2(3H)-one

**Compound 14.**

4-amino-1-benzyl-6-(trifluoromethyl)-1H-imidazo[4,5-c]pyridin-2(3H)-one

**Compound 15.**

5-benzyl-3-(trifluoromethyl)-5H-imidazo[4,5-c]pyridazin-6(7H)-one

**Compound 16.**

4-amino-1-(4-fluorobenzyl)-6-(trifluoromethyl)-1H-imidazo[4,5-c]pyridin-2(3H)-one

**Compound 17.**

4-amino-6-(4-methyloxazol-2-yl)-1-((6-methylpyridin-3-yl)methyl)-1H-imidazo[4,5-c]pyridin-2(3H)-one

**Compound 18.**

4-amino-1-benzyl-6-(2-fluorophenyl)-1H-imidazo[4,5-c]pyridin-2(3H)-one

**Compound 19.**

4-amino-1-(3-fluorobenzyl)-6-methyl-1H-imidazo[4,5-c]pyridin-2(3H)-one

**Compound 20.**

methyl 4-amino-1-benzyl-2-oxo-2,3-dihydro-1H-imidazo[4,5-c]pyridine-6-carboxylate

**Compound 21.**

4-amino-1-(4-bromobenzyl)-6-(trifluoromethyl)-1H-imidazo[4,5-c]pyridin-2(3H)-one

**Compound 22.**

4-amino-1-(4-fluorobenzyl)-6-methyl-1H-imidazo[4,5-c]pyridin-2(3H)-one

**Compound 23.**

4-amino-1-benzyl-6-cyclopropyl-1H-imidazo[4,5-c]pyridin-2(3H)-one

**Compound 24.**

6-amino-9-benzyl-2-(2-methoxyethoxy)-9H-purin-8-ol

**Compound 25.**

4-amino-6-((2-methoxyethylamino)methyl)-1-phenyl-1H-imidazo[4,5-c]pyridin-2(3H)-one

**Compound 26.**

4-amino-6-isopropyl-1-phenyl-1H-imidazo[4,5-c]pyridin-2(3H)-one

**Compound 27.**

4-amino-1-phenyl-6-(pyridin-2-yl)-1H-imidazo[4,5-c]pyridin-2(3H)-one

**Compound 28.**

4-amino-1-(2-methoxyethyl)-6-(trifluoromethyl)-1H-imidazo[4,5-c]pyridin-2(3H)-one
